# Factors Driving High-Need High-Risk Vulnerable Veterans Use of Outpatient Healthcare

**DOI:** 10.1093/geroni/igab046.3601

**Published:** 2021-12-17

**Authors:** Ali Vaeli Zadeh, Fei Tang, Carlos Gomez, Luci Leykum, Orna Intrator, Bruce Kinosian, Willy Marcos Valencia, Stuti Dang

**Affiliations:** 1 Miami VA Healthcare System, Miami, Florida, United States; 2 Miami Veteran Affairs Healthcare System, Miami, Florida, United States; 3 South Texas Veterans Health Care System, San Antonio, Texas, United States; 4 Canandaigua VA Medical Center, Canandaigua, New York, United States; 5 Philadelphia VAMC, Philadelphia, Pennsylvania, United States; 6 Medical University of South Carolina, Miami, Florida, United States; 7 University of Miami, Miami, Florida, United States

## Abstract

Using predictive analytic modeling, the Veterans Affairs (VA) Geriatrics and Extended Care Data Analysis Center (GECDAC) identified vulnerable “High-Need High-Risk” (HNHR) Veterans, as requiring more support and services. We sought to identify variables linked with utilization of our outpatient HNHR C4 clinic offering Comprehensive Geriatric Assessment, Care Planning, Care Coordination, and Co-management". Of 724 HNHR Veterans contacted, 531 were reached and invited to participate; 193 were not reached, 326 were reached but declined the C4 clinic, 205 attended the clinic. We compared these groups. Independent variables were organized using Anderson’s behavioral model into predisposing (age, gender, race, ethnicity), enabling (drive time, service eligibility, Area Deprivation Index, marital status), and need factors (mental health cognitive condition, ambulatory care sensitive conditions, NOSOS, JFI, CAN, etc.). C4 enrollment acceptance was the outcome. Results showed that compared to patients who declined, HNHR veterans who attended C4 clinic had more chronic health conditions(p<0.01), more service eligibility(p=0.01), more driving time to the closest VA clinic(p=0.01), and more were married (p=0.01). Patients who declined C4 clinic might have greater barriers to care access. Accessing needed healthcare among HNHR older adults maybe impacted more by enabling factors that allow the individual to seek care if needed and are the resources that may facilitate access to services, rather than need factors, which include individuals' perceptions of their health and functional state, and healthcare needs assessed by professionals. More social and intermediary determinants of health should be incorporated as enabling factors into models striving to understand drivers of healthcare use.

